# Selecting Treatment Options and Choosing Between them: Delineating Patient and Professional Autonomy in Shared Decision-Making

**DOI:** 10.1007/s10728-019-00384-8

**Published:** 2019-09-21

**Authors:** Emma Cave

**Affiliations:** Durham University, Stockton Road, Durham, DH1 3LE England UK

**Keywords:** Autonomy, Informed consent, Medical law, Montgomery, Negligence, Shared decision making

## Abstract

Professional control in the selection of treatment options for patients is changing. In light of social and legal developments emphasising patient choice and autonomy, and restricting medical paternalism and judicial deference, this article examines how far patients and families can demand NHS treatment in England and Wales. It considers situations where the patient is an adult with capacity, an adult lacking capacity and a child. In all three cases, there is judicial support for professional autonomy, but there are also inconsistencies that have potential to elevate the importance of patient and family preferences. In combination, they may be perceived by healthcare professionals as an obligation to follow patient preferences, even where doing so conflicts with other professional obligations. It is argued that a more nuanced approach to shared decision-making could help clarify the boundaries of decision-making responsibility.

## Introduction

In common with many countries, the UK has enhanced patient choice of provider [27] and has provided internet-based information to aid them in their choices. Can patients also choose what treatments to have? The doctrine of informed consent was recognised in *Montgomery v Lanarkshire Health Board* (*Montgomery*) [54], a case that separates the selection of treatment options, which is under healthcare professional (HCP) control, and the informed choice between them by patients. This paper will show that the spheres overlap and that patient values are relevant to both. It explores how this phenomenon weakens professional control in the selection of NHS treatment options in England and Wales, considers some of the implications and suggests ways of mitigating the effects through differential models of shared decision-making (SDM). Analysis focuses on the balance between patient and professional autonomy in the selection of available treatment options, rather than any public law claim patients might have for access to treatment.

Part I defends the practical and political value in maintaining a degree of patient-focussed professional control over the availability of treatment options. It considers the influence of *Bolam* [18], which established a standard of care in negligence based on medical judgement and protected HCP autonomy. Since *Bolam*, judicial deference to HCPs has waned. In 2015, Lords Kerr and Reed in *Montgomery* stated: ‘social and legal developments … point away from a model of the relationship between the doctor and the patient based upon medical paternalism [57]’. Part I sets out normative arguments for balancing new emphasis on patient choice with professional control in the selection of treatment options.

Part II engages in conceptual analysis to articulate the protection of professional autonomy with respect to three different patient groups: patients with capacity, patients who lack capacity but whose past or present wishes are ascertainable, and patients who lack capacity and whose wishes cannot be determined. In each case, it sets out current protections of professional autonomy and highlights weaknesses that leave them vulnerable to misinterpretation in law and practice.

Part III advances a normative argument as to how decisions across different patient groups might be shared between professionals and patients and argues that it might be applied to the distinction between selection and choice of treatment options. This could potentially be utilised by the judiciary to enhance conceptual consistency and by professional bodies as a practical decision-making tool. The proposed scheme builds on Sandman and Munthe’s models of SDM [81]. Applying particular SDM models to particular patient groups can help delineate the boundaries of decision-making responsibility.

The article’s aims are explanatory and also revisionary in so far as it calls for greater clarity as to the scope of professional autonomy. There is a vast literature embracing SDM and patient autonomy, but its impact on professional autonomy is under-explored.

## Professional and Patient Autonomy

The legal emphasis on patient autonomy in healthcare decision-making has focussed predominantly on patient rights to make an informed choice between treatment options [54]. The selection of the relevant treatment options from which patients choose, meanwhile, has been the purview of HCPs. For Sulmasy, professional autonomy in the selection of treatment options is justified by virtue of their specialised knowledge deployed for the service of patients [84]. The arguments for professional control extend to those cases where patients request treatments or interventions: undue interference from patients, managers, family members or the law courts might result in substandard decisions. McAndrew adds that maintenance of trust in the profession also requires limitations on the adherence to patient choice of treatment:

[I]f physicians are measured on whether or not they comply with all patient requests rather than on what the physician thinks in her professional judgement is the best course of treatment for the patient, what reasons is there to trust the physician’s judgement [52]?

Brazier argues that, whilst patient choice may be a relevant factor in the deployment of HCP discretion around treatment selection, autonomy does not entitle patients to receive any treatment they desire [21]. Montgomery frames ‘”objection” as a key professional role [whereby] the professional is expected to object to “inappropriate” access to the treatments sought [53]’. This flows from recognition that the moral function of HCPs is not merely to serve patient choice, but to protect patients’ wider interests. The normative arguments in support of professional autonomy in the context of treatment selection are reflected in international guidance from the World Medical Association, which requires freedom from undue external interference and ‘supports physicians if they refuse demands by patients and family members for access to inappropriate treatments and services [87]’.

A failure to honour the caring, responsible and competent view of the HCP can impact on resources and exacerbate consumerism if it leads to treatment based on patient preferences, rather than evidence. It can lead to the administration of tests and treatment that are not professionally justified and that expose patients to harm. It can also lead to moral distress amongst HCPs if treatment conflicts with professional values. Moral distress was first described by Jameton [46]. It applies when HCPs have a responsibility to act (for example, by objecting to patient treatment choices that are not professionally justified) but cannot do so in a way that preserves their integrity. One source of it, and what can make it ‘moral’, is the requirement to work in opposition to professional obligations.

There are therefore good reasons for assigning the role of selecting relevant treatment options to HCPs and in limiting the right of patients to demand treatment that HCPs consider inappropriate. Lord Phillips MR in *Burke* supported this position, stating:

Autonomy and the right of self-determination do not entitle the patient to insist on receiving a particular medical treatment regardless of the nature of the treatment. Insofar as a doctor has a legal obligation to provide treatment this cannot be founded simply upon the fact that the patient demands it. The source of the duty lies elsewhere [72].

Lord Phillips asserted that patient autonomy is not of itself a sufficient reason to uphold patient choices but acknowledged the existence of potential sources of a duty to treat. Where a duty to treat can be established, professional autonomy in treatment selection is necessarily limited. It will be argued that patient autonomy has been influential in the development of the sources of the duty to treat.

One potential source of the duty lies in criminal law. For example, cases involving the continuation of treatment in circumstances that HCPs consider burdensome to the patient and futile, might result in the court declaring that continued treatment is in the patient’s best interests. In such cases, there may be a duty to keep the patient alive. Another possible source of duty is negligence. This might apply when an option is not offered that ought reasonably to have been offered, or a patient requests an option that is unreasonably withheld. The 1957 *Bolam* decision [18] set out the standard of care in negligence cases involving skilled professionals. McNair J directed the jury: ‘[A doctor] is not guilty of negligence if he has acted in accordance with a practice accepted as proper by a responsible body of medical men skilled in that particular art’. The test emphasised professional autonomy by making clear that HCPs would not be found negligent if peers would view the practice as acceptable and responsible, notwithstanding that some competent professionals would have acted differently. The potential for judges to prefer one body of evidence from another was limited and HCPs gained considerable powers to determine the standard of care. This deferential position was restricted when the House of Lords asserted in *Bolitho* [19] that a body of expert evidence cannot be relied upon if it cannot be logically supported.

Holm and Devaney have described how changes in the social context in the 1990s emphasised patient choice, patient autonomy and evidenced-based medicine [28]. A failure to honour the wishes of the patient or family limits their choice, the exercise of their autonomy and can impact on their dignity. Social changes influenced legal developments, particularly in relation to information disclosure and best interests decision-making, which will be shown in the next sections to have impacted on the role of professional expertise. Whilst the courts will not order an HCP to treat, the source of a duty to treat may lie in criminal law or the law of negligence. The latter has expanded since the 1990s to enhance patient autonomy.

## Professional Autonomy Across Patient Groups

The judicial interpretation of the balance between professional and patient autonomy in England and Wales has evolved according to patient type. This section considers three such groups, contending in each case that the law maintains a precarious hold on the protection of professional autonomy in the selection of treatment options. Part III will outline a scheme by which it is proposed that the claims could be better accommodated and managed.

### Patients with Capacity

Whereas patients with capacity were once reliant on HCPs to set out the options and make recommendations, many patients now seek out information from additional sources, including the internet [57]. This can lead to requests for interventions that HCPs do not consider justifiable. Whilst the Court of Appeal in *Burke* [72] made clear that adult patients with capacity cannot demand intervention, the extent of the protection this affords to HCPs needs to be reassessed in light of a distinction made in the Supreme Court decision of *Montgomery.* Lords Kerr and Reed differentiated between the HCP’s selection of treatment options, the reasonableness of which is still governed by *Bolam* and *Bolitho*, and the disclosure of those options so that patients can make an informed choice between them, which is now governed by *Montgomery* [31 and see 59]. Different standards of reasonableness are used to assess breach of the duty of care in each case: the *Bolam* standard is a standard of the reasonable HCP, whereas the *Montgomery* standard considers the position of the reasonable, and where relevant the actual, patient [60]. Broadly speaking, the *Bolam* standard emphasises professional autonomy, and the *Montgomery* standard prioritises patient choice and autonomy: Lords Kerr and Reed said that ‘patients are now widely regarded as persons holding rights, rather than as the passive recipients of the care of the medical profession [56]’.

Intuitively, this seems a logical distinction and indeed it fits many treatment situations: HCPs utilise their expertise to establish one or more suitable options. This might include the option of no treatment. If the patient later complains that an HCP’s failure to offer an alternative has caused them harm, the HCP’s actions will be judged according to the *Bolam* standard. A separate matter is for the patient to choose amongst the options. At this point, patients require information on the relative risks and benefits. This matter is personal to the patient and their values. The adequacy of this information is therefore judged according to the *Montgomery* test.

This assumes, however, that the patient’s values will not be relevant to the selection of treatment options. Patients might assert that the failure to offer alternative treatment (or accept their request for it) was unreasonable and has caused them harm. Such a claim would be strongest where the alternative carries less risk than the proposed option/s and is broadly as efficacious. In *Birch* [17], Mrs Birch complained that her doctor was negligent in not informing her of a slightly less efficacious but lower risk diagnostic procedure. Applying *Pearce* [69], Cranston J allowed the claim on the basis that the duty to inform a patient of relevant risks is only discharged if the patient is informed of alternative procedures that carry fewer or no risks. Consider also *Montgomery* itself, where the alternative to vaginal delivery was a caesarean section. In the circumstances, the court held that Nadine Montgomery should have been informed of this alternative.

What if the alternative is less efficacious, but would be supported on the basis of the patient’s values and preferences in circumstances where the HCP ought reasonably to know of them? For example, a suboptimal intervention would have allowed an opera singer to keep singing, or a footballer to keep playing. In both cases, if reasonableness is judged according to *Bolam* rather than *Montgomery,* the patient is less likely to succeed. They might therefore seek to assert *Montgomery*’s relevance.

Three arguments would support the claimant. First, the *Montgomery* decision protects choice [55]. If *Montgomery*’s application is extended to the selection by HCPs of treatment options, then the reasonableness of the HCP’s selection would be judged in the context of the management of the reasonable and particular patient, rather than the reasonable HCP. Professional autonomy would be reduced, patient choice elevated. Second, *Montgomery* pertains to advice. Whilst advice once focussed predominantly on *how* to choose between options selected by HCPs, the practical reality is that it now frequently extends to the suitability of options patients have selected having conducted independent research. Treatment selection is no longer the exclusive domain of the HCP. Third, it is arguable that the basis for the distinction between selection and choice amongst treatment options is out of touch with practice. According to Lady Hale in *Montgomery*, where the consideration of the HCP’s actions departs ‘from purely medical considerations and involves value judgments … *Bolam* … becomes quite inapposite [61]’. To be clear, neither Lady Hale nor Lords Kerr and Reed deny that medical considerations may be *part* of the issue of information disclosure. Rather, they say that once value judgements become relevant, *Bolam* should cease to apply. In the bracket of ‘pure medical considerations’, Lords Kerr and Reed include ‘the doctor’s role when considering possible investigatory or treatment options [58]’.

2001 General Medical Council (GMC) guidance concurred with this position: *Good Medical Practice* guidance required doctors to provide treatment ‘based on your clinical judgement of patients’ needs and the likely effectiveness of the treatment [34]’. In other words, the selection of treatment options was a matter of professional expertise, the reasonableness of which would be judged according to the standard of the responsible, competent doctor. Today, however, GMC guidance that is endorsed in *Montgomery* supports the view that non-clinical considerations *are* relevant to treatment selection. It says that patients should have *information they want or need* around options for treatment [36]. Accordingly:

The doctor uses specialist knowledge and experience and clinical judgement, *and the patient’s views and understanding of their condition*, to identify which investigations or treatments are likely to result in overall benefit for the patient [35].

2018 draft guidance from the GMC makes it explicit that non-clinical considerations are relevant to the selection of treatment options by HCPs:

If a patient asks for treatment or care that you don’t think would be beneficial to them, you should explore the patient’s reasons for requesting it. When assessing any likely benefit, *you should take into account factors that are significant to the patient, including non*-*clinical factors such as the patient’s beliefs or views and the possible effect on their lifestyle* [40].

The *obiter* statements in *Montgomery* about the selection of treatment options being ‘purely medical considerations’ conflict with professional guidance and with the practical reality that today’s patients will sometimes conduct their own research on treatment options and make requests that will sometimes be based on their wishes and value judgments.

If the *Montgomery* test is applied to HCPs’ selection of treatment options as well as their disclosure of information on the risks and benefits of those options, then the effect will be to limit professional autonomy. The assertion by Lord Phillips MR in *Burke* that; ‘[A] patient cannot demand that a doctor administer a treatment which the doctor considers is adverse to the patient’s clinical needs [74]’ would still apply, but it would be easier to demonstrate that a decision to deny a treatment option is unreasonable and therefore negligent. So far, the Scottish courts have not seen fit to extend *Montgomery* to treatment selection [2, 85]. The matter has not been directly raised in the English courts (for further exploration see Cave, forthcoming).

In summary, patients with capacity cannot *demand* medical interventions. HCPs retain considerable control in the selection of treatment options. However, there is a lack of clarity as to the appropriate standard of care by which the reasonableness of that selection will be judged in negligence cases. Additionally, there is evidence of enhanced expectations of patient choice and enhanced patient access to information. These factors have potential to impact on professional autonomy in the selection of treatment options. Dialogue between the HCP and patient will often resolve disputes as to what treatment is appropriate, but desperation, hope, lack of trust, or a consumeristic assumption of the right to choose might lead to patient dissatisfaction with the range of options available. Even if the arguments set out in this subsection do not lead to legal change (and there are good reasons why they should not), there is evidence of their impact in practice. Some HCPs are sanctioning options that the evidence-base does not support [31]. This, in turn, could potentially result in moral distress for the team of HCPs required to administer the treatment.

### Patients Lacking Capacity Whose Wishes Can be Ascertained

Turning now to patients who lack capacity, Article 12 of the United Nations Convention on the Rights of Persons with Disabilities 2006 (CRPD) requires equal recognition before the law, including respect for the rights, will and preferences of the person. The CRPD is an international legal agreement signed by the UK in 2009. It has been influential in common law development, protecting the autonomy rights and interests of people with disabilities. This section begins by charting the development of the legal approach in cases where the HCP view of what treatment is appropriate conflicts with evidence of the wishes and preferences of patients. A relative reduction in professional autonomy is demonstrated. This is balanced by limits imposed on judicial powers to order individual HCPs to treat contrary to their judgement, but flaws in these controls are exposed.

In the 1990s and early 2000s, *Bolam* was applied beyond the law of negligence to bridge gaps in law and professional guidance. In *Re F* [32], *Bolam* was applied to determine what treatment options were in the best interests of a patient lacking capacity [20]. This protected professional autonomy when patients or family members requested treatments that HCPs considered inappropriate. The courts would not countenance requiring HCPs to treat contrary to medical opinion. In *Re J* [1993], Lord Donaldson MR stated:

I cannot … conceive of any circumstances in which this would be other than an abuse of power as directly or indirectly requiring the practitioner to act contrary to the fundamental duty which he owes to his patient [45]. (Note approval in [3].)

Lord Donaldson’s refusal to ‘indirectly’ require an HCP to act implies that the court would not hold that an HCP, acting on the basis of professional obligations, was acting contrary to the best interests of the patient. Today, it remains the case that the courts will not directly require an HCP to treat a patient contrary to their professional view, but there is less reluctance to indirectly require action by the NHS Trust. The court can conclude that the HCP view of what option is in the patient’s best interests is erroneous.

The change in position came about gradually. The deferential position adopted in *Re F* was restricted by Butler Sloss P in *Re S* [20, 80]. It was opined that *Bolam* gives room for reasonable professional disagreement because there may be more than one acceptable medical opinion, whereas the best interests test required that the best option be found. A ‘balance sheet approach’ was developed [1], that allowed potential gains and losses to be assessed in order to find the preferable route. Enacted in 2007, the Mental Capacity Act 2005 (MCA) established the Court of Protection (CoP), which has wide powers under section 15(1) (c) to make declarations as to ‘the lawfulness or otherwise of any act done, or yet to be done, in relation to [the] person’. Best interests are now considered in the widest possible sense to include not only clinical, but social and psychological factors [4]. In making best interests determinations, the court’s role is to find the best option in light of P’s values [5]: it is an objective test from the patient’s point of view [26]. Section 4(6) of the MCA requires weight to be given to the ‘past and present wishes and feelings’ of the incapacitated person, and the ‘beliefs and values that would be likely to influence [their] decision if [they] had capacity’. The courts have interpreted section 4(6) to enhance parity between those with capacity and those who lack capacity and whose wishes can be ascertained [26].

Evidence that a course of action fits with the patient’s preferences is therefore a relevant factor when HCPs make best interests decisions according to the section 5 MCA general legal authority. It is also relevant when the court makes declarations under section 15. HCPs may seek assurance from the court that they are correct in their professional view that a treatment option requested by family or the patient is contrary to the patient’s best interests and should bring an application in cases of disagreement [67]. There is potential to breach the rights of patients by failing to bring disputed cases before the court [41]. A court declaration can conflict with the HCP view, as is clear from two recent case examples. In *University Hospitals Birmingham NHS FT v HB* [86], HB was 61 when she collapsed having suffered a cardiac arrest and irreversible hypoxic injury with evidence of brain stem dysfunction. The Trust applied to withhold six potential treatments, set out in a plan. The Trust argued that the specified treatments were burdensome to HB and lacked the prospect of allowing her to resume a meaningful quality of life. Keehan J acknowledged that the balance of medical evidence supported the view that the treatments were futile and burdensome but looked beyond the clinical assessment of best interests. FB gave evidence that her mother, a practising Muslim, would want all possible steps to be taken to keep her alive. Applying the balance sheet approach, Keehan J decided that the patient’s wishes justified the treatments set out in the plan, although he accepted that this might change in due course.

A comparable case is *Royal Bournemouth and Christchurch Hospitals NHS Trust v TG & OG* [76]. TG was in a vegetative state following a subarachnoid haemorrhage which resulted in severe cerebral dysfunction. The medical view was that it was not in TG’s best interests to continue with intubation [77]. Applying section 4 of the MCA, ‘to which the individuals wishes, feelings, beliefs and values are central feature [78]’, Cohen J held that intubation should continue. As in *HB*, the issues were finely balanced: Cohen J was asked to make the decision a mere two months post injury and before vegetative state can be regarded as permanent [79]. Cohen J acknowledged that the House of Lords in *Airedale NHS Trust v Bland* [6] had recognised that it was not in the best interests of a patient in a vegetative state to undergo futile treatment, but argued that the law has moved on: *Bland* needs to be considered on its facts and in light of its pre-MCA status. TG’s Catholic faith was of huge importance to her. The evidence suggested that she would want to keep living. Cohen J concluded:

[30]. I recognise that this places a huge burden on the treating team. It is against their advice and their wishes … but I remind myself constantly, this is her life and her wishes as I have found them to be and nobody else’s.

*HB* and *TG* are powerful reminders that the HCP should accommodate the wishes and preferences of patients when making best interests assessments. More pertinent to this paper, however, is that whilst the court will not order an individual HCP to treat contrary to professional judgement, a best interests determination can trigger a collective duty to do so. This constitutes an expansion of judicial powers when contrasted with the refusal in *Re J* to ‘directly or indirectly’ require HCPs to act. Individual HCPs can still refuse to treat on the basis that doing so would conflict with their individual conscience [38, 90]. GMC guidance states that: ‘The law does not require doctors to provide treatments or procedures that they have assessed as not being clinically appropriate or not of overall benefit to the patient [37].’ However, in some circumstances, this will only offer a solution is someone else is willing to treat [21].^1^ Withdrawal of care would potentially constitute a breach of duty and, if the patient were to die, could leave the HCP open to the charge of murder or gross negligence manslaughter. Furthermore, individual conscience is poorly defined and safeguarded, particularly in non-religious cases where it is the situation rather than a particular procedure (such as abortion) to which the HCP objects [83].^2^ A collective duty does not *require* individual HCPs to treat, but the reality is that it will sometimes put them under considerable pressure to do so.

The courts have made efforts to ensure that the reductions in professional control described above are not expanded. Four limits on their powers have been articulated. First, the Supreme Court made clear in *Aintree* [3] and *N v ACCG* [62] that the court can do no more for a patient who lacks capacity than it could if the patient had capacity. Wishes and preferences do not justify treatment that capacitous patients would have no right to demand. Second, Lady Hale has reiterated the position set out in *Burke* [74] that the patient (and therefore the court) ‘cannot order a doctor to give a particular form of treatment [3]’ (and see [12, 64]), and cannot order a Clinical Commissioning Group to fund treatment patients or family desire [75]. Third, Lady Hale in *Aintree* stated that if ongoing treatment of patients lacking capacity does not comply with the patient’s best interests, ‘it will follow that it will not be lawful to give it [4].’ This means that preferences for ongoing treatment are subject to a best interests determination. Finally, the court will not address hypothetical or ‘academic’ matters, in which category it places treatment sought by a patient or family, which no HCP is willing to offer. In *Re J* [1991], Lord Donaldson MR opined:

What the court can do is to withhold consent to treatment of which it disapproves and it can express its approval of other treatment proposed by the authority and its doctors [44].

Lady Hale referred to this passage in *Aintree* [3]. It implies that, whilst HCPs can seek declaratory relief that has potential to go against the medical view, patients and family can only do so if they can locate an HCP willing in principle to treat. In the 2011 case of *AVS (by his litigation friend CS) v An NHS FT* [11], the brother of a patient with sporadic Creutzfeldt Jakob’s Disease sought a declaration that an additional course of novel treatment was in the patient’s best interests in circumstances where the patient’s current doctor was unwilling to treat. The brother had medical evidence to support his case but was unable to file a report stating that another consultant neurologist was willing and able to take over the care of the patient and that the treatment was in the patient’s best interests. The application was refused: ‘if there is no one available to undertake the necessary operation, the question of whether or not it would be in the patient’s best interests for that to happen is wholly academic [13]’.

Despite these powerful safeguards, there are risks that HCP autonomy might be further eroded. In the context of HCP best interests determinations under section 5 MCA, the practical impact of *Aintree* may be that HCPs become less willing to contradict the wishes and preferences of patients lacking capacity, even if the evidence-base does not support treatment. In 2017, the Law Commission proposed that powers under section 5 be constrained where they involve interference with the rights of individuals under article 8 of the European Convention on Human Rights [51] which incorporates self-determination [71]. The Law Commission also proposed revisions to section 4(6) MCA to give increased weight to wishes and feelings [50]. In the context of court declarations, the limits imposed on the court are significant, but it seems that one of them at least might be subject to challenge. Since *AVS*, protection of patient wishes and preferences has expanded and deference to HCPs limited. A challenge to *AVS* could build on the fact that not all hypothetical cases are barred from judicial consideration. In *Wyatt* [70], the Court of Appeal held that the hospital was correct to approach the court before the anticipated developments in the child’s condition occurred. Whilst the court should avoid making open-ended declarations they might need to revisit if circumstances change, some questions need to be dealt with in advance. The hypothetical nature of the case is therefore fact and case specific. In the 2017 case of *N v ACCG*, Lady Hale confirmed that the CoP is entitled to take the view that ‘no useful purpose’ is served by hearing an issue [63]. But given that the CoP can challenge and refute the HCP view of best interests, it is arguable that the judgment *would* serve a useful purpose: it would consider whether to impose a duty on the Trust (rather than any individual) to treat. AVS raised a hypothetical question because no doctor had agreed to treat, but a willing HCP might well be found if the court determines that treatment is in the patient’s best interests, thereby establishing a collective duty to treat.

Consider a fictional scenario: P’s family seek novel treatment, which treating HCPs do not consider compatible with P’s best interests or their professional obligations. According to *Aintree*, the burdensome nature of the intervention and evidence of a low chance of success is not a bar if P’s past or present wishes and preferences favour treatment. According to the recent case of *B v D* [16], some degree of risk relating to novel treatments should not preclude access for an incapacitated patient who has demonstrated a strong wish to proceed. Lack of resources might justify refusal—the National Institute for Health and Care Excellence is tasked with deciding what treatments the NHS will fund—but some patients are self- or crowd-funded. It is conceivable that, on the application of the balance sheet approach, the treatment could be declared to be in the best interests of an incapacitated patient, even if the HCPs giving evidence are opposed to the treatment. Those HCPs would not be required to treat, but the Trust would be under an obligation to find someone willing to treat given the court’s finding that such treatment is in the patient’s best interests. The contrary position of refusing to hear the case because it is hypothetical until a willing HCP is found is deferential to HCPs, because it allows them to define the best interests of the patient. It raises issues of access to justice if incapacitated patients, notwithstanding medical evidence to support their case, are denied the opportunity for the court to weigh their psychological and emotional interests alongside their clinical interests when determining best interests.

It is not argued here that extending the powers of the court to consider hypothetical options would be a positive development. Rather, the purpose is to show that further reductions in professional autonomy might flow from the combination of several factors. These include the analogical development of the focus on the wishes and preferences of the patient, combined with the willingness to use declaratory powers to contradict the HCP view of best interests, which can indicate a collective duty incumbent on HCPs to treat in circumstances where they consider it contrary to the patient’s interests. The judiciary could helpfully acknowledge the effects of best interests decisions that indirectly result in a collective duty to treat, so as to take into consideration public policy arguments against its expansion.

### Patients Who Lack Capacity and Whose Wishes Cannot be Ascertained

Where a patient over the age of 16 lacks capacity and their past and present wishes cannot be ascertained, the HCP will consult with relevant others where practicable, to determine which of the clinically relevant options is in the patient’s best interests. Where the patient is under the age of 16 and lacks capacity, the MCA does not apply. A proxy will provide consent in non-emergency situations and they will choose between the options selected by the HCP. For one group of patients in particular, there is a potential challenge to HCP control over the selection of treatment options. This emanates from claims of those with parental responsibility (for whom the shorthand ‘parents’ will be used) to a right to choose medical treatment for their young children.

Disputes about children are heard in the family court which considers the best interests of the child in line with the paramountcy principle set out in section 1(1) of the Children Act 1989. The family court will not require individual HCPs to treat, but (as discussed above in relation to the Court of Protection) by making a best interests determination, a collective duty can be triggered [14]. For example, in *An NHS Trust v MB* [8], Holman J held that ventilation of an 18-month-old baby with severe spinal muscular atrophy should not be withdrawn, contrary to the view of the Trust. Holman J did not order a medical intervention against the judgement of HCPs. Rather he declared that it was in MB’s best interests for existing treatment to be prolonged. A duty was established to keep the patient alive.

The duty is collective. The impact on individual clinicians will depend on the availability of other clinicians who are willing to treat. Hedley J in *Re Wyatt* [88] recognised that whilst the professional duty of HCPs is to act in the best interests of the patient, HCPs should not be required to treat if to do so would be ‘an affront to conscience [89]’. However, the HCP ‘should not prevent another from so acting, should that clinician feel able so to do [90]’. Dame Butler-Sloss went further in *B v An NHS Hospital Trust,* in the context of a treatment refusal by an adult patient with capacity, stating: ‘If there is no disagreement about competence but the doctors are for any reason unable to carry out the wishes of the patient, their duty is to find other doctors who will do so [15].’ Where the court contradicts the medical view of what treatment is in the best interests of the child patient, individual HCPs will not be required by the court to carry out the treatment but may come under a professional duty to act if others are not willing to treat. Additionally, two other situations may arise in which individual HCPs may be required to treat contrary to their view of the patient’s best interests.

One flows from the practical requirements of procedural justice (fair processes), which may require treatment that both the lower courts and HCPs maintain is contrary to the child’s best interests. The case of Charlie Gard is a case in point [42]. Charlie was diagnosed with mitochondrial DNA depletion syndrome. HCPs considered further treatment, including unproven nucleoside therapy in the USA, to be contrary to his best interests in light of its futility and potential to prolong suffering. They applied to withdraw life support and provide palliative care. The parents’ appeal of the High Court decision [90] was rejected by the Supreme Court and the European Court of Human Rights. During the lengthy appeals process, HCPs were collectively obliged to continue to treat Charlie in circumstances where the lower courts acknowledged that treatment was contrary to his best interests. Whilst efforts might be made to speed up the judicial process, it is difficult to envisage a way to avoid the breach of professional conscience and resulting moral distress that results. Emphasis in such cases must be on enhancing resilience through training and support of HCPs [29].

A second issue arises in the form of increased pressure to acknowledge a parental right to choose treatment that does not cause significant harm to the child, notwithstanding that HCPs consider the treatment to be contrary to the child’s best interests. In *King* [47], parents objected to the treatment proposed by the hospital for their child, Ashya, and took him abroad for proton beam therapy. They were arrested due to concerns for the child’s safety. A week later, Baker J found that the situation had changed. The local authority and Child and Family Court Advisory and Support Service on behalf of Ashya did not object to the parental plans for treatment and nor did the NHS Trust oppose the plan in principle. Baker J said that the court:

has no business interfering with the exercise of parental responsibility unless the child is suffering or is likely to suffer significant harm as a result of the care given to the child not being what it would be reasonable to expect a parent to give [48].

In *Gard*, counsel for the parents argued that the significant harm threshold should apply in limited situations where parents propose an alternative treatment, have found a clinician willing to treat, and have secured the necessary resources (in this case, through crowd-funding). The Court of Appeal rejected the argument [91]. The harm test had relevance in *King* because the initial judicial intervention was based on failure to treat rather than a dispute as to which treatment option was in the child’s best interests [9]. In *Gard* it was found that the case law is clear: *NHS Trust v MB* establishes that, once the case comes before the court, the best interests standard applies [66]; In *Burke*, Lord Phillips MR said: ‘Once a patient is accepted into a hospital, the medical staff come under a positive duty at common law to care for the patient [73].’; In *Re F* [33], Lord Brandon of Oakbrook confirmed that the duty is to act in the patient’s best interests. In *Gard*, McFarlane LJ affirmed that the best interests test applies. Parental views are relevant to best interests, but not determinative [92].

The position taken in *Gard* that the best interests test rather than the significant harm test applies, has proved contentious. Following two further cases involving treatment disputes relating to seriously ill babies [7, 49], a legal campaign has been mounted to advance parental rights [24, 43]. Auckland and Goold argue that parents should be able to choose between available medical options, yielding authority only when their decision carries a serious risk of significant harm [10]. I have previously expressed concern as to the implications for children’s rights of extending the significant harm test [22], but what is pertinent to this article is that, if the argument were successful, it would narrow the scope of professional autonomy in two ways. The first is relatively subtle. If application of the harm threshold is limited to situations where a parent seeks to move the child to a willing HCP in another country, NHS clinicians will not be required to treat contrary to their view of best interests. As Archard points out, however, their professional autonomy is still affected:

Even if there is an irresolvable disagreement of values between doctors and parents–irresolvable because the respective values cannot be measured against one another—it is far from clear why we should decide in favour of the parents. It is said that so deciding would be ‘unfair’ inasmuch as it would mean imposing one set of values—presumably those of the doctors—on the parents. Yet, why would it not be unfair to impose the parents’ values on the doctors? After all they, as doctors, have a professional duty to do what they see as best for their patients, and they could not discharge that duty as they understand it if they must do what they do not believe to be the best for their patient [9].

Furthermore, it is questionable that the harm threshold could be so confined. If it is based on the right of parents to have their values considered to an extent that they do not pose a serious risk of significant harm to their child, then they might claim that their rights and values are also relevant to HCP decisions to discontinue treatment where continued treatment would not cause significant harm. Incrementally, parents might also argue that consideration of their values requires offering treatments that, whilst not in the child’s best interests, do not pose a serious risk of significant harm. If so, they could not demand such treatment, but they might have a stronger claim that it might reasonably be offered, breach of which could potentially give rise to a claim in negligence. In that case, the effect would be that parental rights to demand treatment gain ground.

## Shared Decision-Making

So far, Part I has set out some of the risks associated with further reductions in professional control of treatment selection, and Part II has shown how they might come about in three contexts. In isolation, each claim of legal ambiguity invites straw man refutations. After all, the courts have made efforts to balance patient and professional autonomy. Lady Hale in particular, is a paragon in this regard. Cumulatively, however, they comprise a precarious balance of professional and patient autonomy. Two risks flow from the legal ambiguities highlighted. One is that the lower courts might fail to adequately protect professional autonomy to select treatment options, in order to enhance the autonomy interests of patients and parents. To guard against this, the judiciary might consider emphasising the need for and value of professional autonomy, recognising the effects of decisions that conflict with medical opinion on HCPs, articulating the scope and application of conscientious objection and addressing areas of doubt referred to in Part II.

The second risk is more practical in nature. Across all three patient groups, there is evidence of opacity as to the boundaries of professional and patient autonomy. HCPs can misconceive the powers and rights of patients, family or parents to select or demand treatment, so that the duty to share decision-making may sometimes be perceived as an obligation to follow patient preferences. Epstein has presented evidence of treatment in contravention of the evidence base [31], and the CMO for Scotland’s 2018 report calls for ‘measures to reduce over-medicalisation and defensive medicine [25]’: measures that can be stymied by ‘the worry that professionals will be criticised for not offering all available treatments and doing all that can possibly be done for each and every patient [25]’.

This section proposes a mechanism by which uncertainty might be reduced. It is argued that, by giving more attention to the *dynamics* of decision-making, a better and more nuanced combination of patient preferences, professional expertise and the evidence-base might be achieved. Patient choice and paternalism are both unilateral models whereby one party holds the decision-making powers [23]. A more personalised approach to choices in medicine involves shared decision-making (SDM). Principle 1 of draft 2018 guidance on consent from the GMC requires doctors to work in partnership with patients [39], but available guidance says little about how SDM might differ across patient groups and decision-types.

Sandman and Munthe [82] argue that, within the term ‘SDM’, there are in fact several different versions which put varying emphasis on patient and professional autonomy. This section agrees with Sandman and Munthe that where SDM is required (by law or professional guidance), more precision is needed as to the relevant SDM model. It further argues that better articulation of SDM would aid the management of patient and HCP expectations. What is proposed in this section is that aspirational SDM models are developed for different patient groups.

### Sandman and Munthe’s Models of SDM

Sandman and Munthe [82] have shown that SDM can retain a shared element whilst emphasising different values. Developing work by Charles et al. [23], they argue that patient choice and paternalism are not necessarily in conflict. They set out nine variations of SDM across a scale from shared-paternalistic to shared-patient-choice-centred models. Four of these require ‘high-level dynamics’. By this, Sandman and Munthe mean that they go beyond simple information sharing, to involve discussion and evaluation. In doing so, the four models provide a tool for conflict resolution, enhance rational discourse and improve the HCP-patient relationship. On this basis, they argue (and I accept) that there is normative value in high-level dynamics.

Focusing on these four models, (i) Shared Rational Deliberative (SRD) Patient Choice and (ii) SRD Paternalism emphasise patient choice and paternalism respectively in relation to formal control. Both emphasise active participation of the patient as a reasoning (rather than an informed or informing) party. (iii) SRD Joint Decision-Making requires deliberation on more equal terms in order to bring about a joint decision, which cannot be reduced to paternalism or patient choice. This model is not simply a matter of crude negotiation, in part because the HCP is constrained by the professional and ethical goals of health care [82]. SRD Joint Decision-Making emphasises rational deliberation in the hope of achieving consensus. Where reaching the best possible joint decision proves impossible, formal control can be asserted in line with the SRD Patient Choice or SRD Paternalism model, depending on value preferences. Rather than fall back on SRD Paternalism, a fourth model called (iv) Professionally Driven Best Interest Compromise is relevant. In this model, the goal is an *effective* decision, which, whilst not rationally preferable, is one with which the patient would adhere. It is a paternalistically driven strategic compromise, that may involve persuasion of the patient. The HCP compromises, within the limits of professional boundaries, with the aim of protecting the patient’s best interests, which incorporate both patient autonomy and the value in an enduring HCP-patient relationship.

Because SDM can be interpreted in different ways, it is important to define which models are applicable in different situations. Sandman and Munthe set out a normative argument as to which is the ideal model of decision-making in healthcare. This section does not engage in that debate. Instead, it considers the relevance of the models in the practical articulation of the legal framework in England and Wales, in the limited context of the distinction between treatment selection and treatment choice.

### Reducing Ambiguity Through SDM

In relation to adults with capacity, the decision in *Montgomery* emphasises dialogue aimed at facilitating patient choice. The optimal expression of this decision, I would argue, is the SRD Patient Choice model (model i). Sharing, rationality and deliberation are not prescriptions for patient choice, but ideals by which HCPs can usefully engage with the patient. Some patients will choose to rely more extensively on the medical view of which option is best.

In the selection of treatment, on the other hand, the implication in *Montgomery* is that the HCP makes a unilateral decision, but we have seen that professional guidance recommends elements of SDM. SRD Paternalism (model ii) requires the authorisation of both parties, which in turn requires rational or autonomous participation, voluntariness and information. The HCP makes the decision but having deliberated with the patient to evaluate their views and preferences. This will be particularly applicable where patients actively suggest or request treatment.

The emphasis in treatment selection is on professional expertise and control, but it is not purely paternalistic as it does not treat the patient as someone whose views, values and preferences are irrelevant to the selection. Professional guidance asserts that the selection of treatment options by HCPs is not merely a technical decision and does not ignore the values of the patient. Recognition that different aspects of SDM are relevant to treatment selection by HCPs and choice amongst options by patients, would both enhance the personalisation of decision-making and reduce the, sometimes artificial, dichotomy between consent and treatment selection. At the same time, it would emphasise the relevance of professional expertise to the treatment recommendations and of patient autonomy in the acceptance of proffered treatment.

Sandman and Munthe focus on patients with capacity who are capable of rational deliberation. Because best interests decision-making involves deliberation, evaluation and, in the context of section 4(6) of the MCA, elements of substituted decision-making, nuanced models of SDM might usefully be developed here too. They could help establish the importance, and also the limits, of patient preferences in relation to selection of and choice between treatment options. Patient preferences should not dictate the selection of treatment options, though they are a relevant consideration. The choice *between* viable options places great emphasis on past and present wishes, but it does not cede control to the patient lacking capacity or their representatives. Best interests remains a balancing exercise in which the patient’s wishes are ‘central feature [78]’.

Where disputes arise as to the treatment options for those lacking capacity, Sandman and Munthe’s Professionally Driven Best Interest Compromise (model iv) has relevance. When the court makes a best interests determination, it aims to find the best option from the patient’s perspective. Only one option can achieve this. Conversely, from a clinical perspective, clinically-suboptimal options that accommodate patient and family preferences might be acceptable. This is because best interests are not determined on clinical factors alone. The HCP must consider the emotional interests of the patient and must look beyond the benefits of treatment to also consider its burdens, which include the impact of the proposed decision. Where a proposed treatment option will result in alienation, coercion or non-adherence to treatment, for example, then the assessment of best interests might alter accordingly. These factors can create room for compromise [22]. In some cases, they will not, because family or HCPs have a line which cannot be crossed. For HCPs this line is governed by laws and professional standards, including the *Bolam* standard of reasonableness and the best interests test. Where compromise would, on their interpretation, involve crossing this line and the dispute cannot be resolved informally, application to the court will be appropriate. This SDM model accommodates parental views and the past and present wishes of the incapacitated adult within the best interests determination, without requiring HCPs to act in a manner that contravenes the patient’s best interests. It creates scope for compromise within the application of the best interests test.

This section has argued for the development of a more nuanced application of SDM. The courts might contribute to its refinement: greater recognition of SDM and its subtle variations might help to resolve some of the legal ambiguities outlined in Part II. For example, the *Montgomery* distinction between treatment and selection would be rendered less stark by acknowledging the relevance of SDM in selection as well as choice of treatment, without risking further encroachment on professional autonomy; the limits of best interests decision-making to protect parental choice and the wishes and preferences of adults lacking capacity could be clarified.

## Conclusion

Social and legal changes have limited judicial deference to HCPs and medical paternalism, and emphasised patient-centred care, autonomy and choice. Professional control of treatment selection has waned in response to *Bolam*’s altered remit, the rise of parental rights and enhanced focus on patient preferences in the best interests test. A balance has resulted in which HCPs have ultimate control of the selection of treatment options and capacitous, informed patients have control of the choice between them. ‘Ultimate control’ does not denote *complete* control. Patient-centred care requires that patient values are relevant in the selection of treatment options, and professional advice is relevant to the choice between them. Where patients lack capacity and their wishes and preferences can be established, the law now gives a degree of parity to patient values [26].

There are strong reasons to maintain patient-centred professional control of treatment selection: it values professional expertise, limits consumeristic tendencies and supports evidence-based medicine. A problem articulated in this article is that the distinction between selection of and choice between options is not always clear in practice and law. A bright line approach which excludes consideration of patient values from treatment selection has been shown to be erroneous and contrary to professional guidance. Conversely, too much emphasis on patient choice can leave HCPs reluctant to object to demands or requests, even when treatment would contradict professional obligations. SDM is required, but it must be responsive to the nuances of the patient group and decision-type.

Three patient groups have been considered: those with capacity, those lacking capacity but whose past and present wishes can be ascertained, and very young patients. In each case it has been argued that opacity or pressure to reform the law blurs the boundaries of professional control of treatment selection. The proposed way forward is not a radical change in law, but a clearer focus on SDM to ensure balance between the values of professional expertise and control, and of patient autonomy in the sense of rational deliberation rather than sheer choice. In relation to capacitous patients, judicial acknowledgement that patient values can be relevant to treatment selection is needed. It is respectfully suggested that a better justification for the *Montgomery* distinction as to the different standards that apply in relation to selection of and choice between treatment is that clinical expertise is *more* relevant to the former, rather than the only consideration. A better justification for reasonable disagreement between parental values and medical considerations of best interests in the case of young children is that there is often room for compromise within the clinical consideration, rather than parental rights to choose options that are not in the child’s best interests. In the case of patients lacking capacity whose wishes can be ascertained, the current and potential effects of the subjective elements of the best interests test on HCPs deserve greater judicial acknowledgement. The CoP will not order an HCP to treat, but this does not mean that HCPs are not collectively required to treat against their view of professional obligations. Acknowledgement would (and should) not prevent this occurring but would raise relevant public policy considerations against incremental expansion.

Professional guidance on SDM is becoming increasingly pervasive across different aspects of care e.g. [39, 65, 68]. The CMO for Scotland’s 2018 annual report, however, recognises that professional guidance can only go so far in delivering SDM. It calls for a change in practice and values to promote ‘realistic’ shared decision-making, that recognises the role and value of both clinical expertise and patient choice. The Report suggests that the focus on ‘consenting’ patients may need to change:

Perhaps working towards a ‘request for treatment’ from patients, rather than a focus on consent forms would help us to move towards a more person-centred decision-making process.

This development would make clearer that whilst SDM is relevant to both treatment selection and choice between treatment, the former is more heavily dependent upon clinical considerations. It would help to manage patient and HCP expectations and delineate responsibilities.

## Footnotes

1 Note that the Conscientious Objection (Medical Activities) Bill [HL] 2017–2019 would enhance protection of HCPs in relation to certain procedures, including withdrawal of life-sustaining treatment. At the time of writing, a decision by four obstetricians at St Luke’s Hospital, Kilkenny, Ireland, ‘that the hospital is not an appropriate location for medical or surgical terminations’ constitutes institutional objection. Patient care will inevitably be affected. See O’Regan, E. ‘’Women will be forced to travel for abortion’ after doctors’ decision’ *The Independent (Ireland)* 24 June 2019.

2 There is a rich literature on the scope and role of conscience which is beyond the scope of this paper.
